# MicroRNA-27a regulates hepatic lipid metabolism and alleviates NAFLD via repressing FAS and SCD1

**DOI:** 10.1038/s41598-017-15141-x

**Published:** 2017-11-03

**Authors:** Meiyuan Zhang, Weilan Sun, Minghao Zhou, Yan Tang

**Affiliations:** Emergency Intensive Care Unit, Qingpu Branch of Zhongshan Hospital, Fudan University, Shanghai, 201700 China

## Abstract

MicroRNAs are implicated as crucial mediators in metabolic diseases including obesity, diabetes, and non-alcoholic fatty liver diseases (NAFLD). Here, we show miR-27a attenuated hepatic *de novo* lipogenesis and alleviated obesity-initiated NAFLD through inhibiting *Fasn* and *Scd1* in liver. Hepatic levels of miR-27a were significantly augmented in HFD-fed and *ob/ob* mice. Further studies demonstrated that miR-27a directly interacted with 3′ untranslated region (3′-UTR) of hepatic *Fasn* and *Scd1* mRNAs and reduced their expression levels in mice. Adenovirus-mediated overexpression of miR-27a robustly blocked sodium oleate-induced triglyceride (TG) accumulation in mouse primary hepatocytes and reduced liver TG contents in mice via repressing hepatic lipogenesis. Furthermore, ectopic expression of hepatic miR-27a impaired lipid contents of livers and attenuated NAFLD development through suppressing lipogenesis in HCD-fed and *ob/ob* mice. Together, our results reveal a critical role of miR-27a in lipid homeostasis of liver and pathogenesis of NAFLD.

## Introduction

Non-alcoholic fatty liver disease (NAFLD), as one of the most common chronic liver diseases worldwide, characterizes by excess lipid accumulation in liver without alcohol consumption. NAFLD encompasses a wide spectrum ranging from hepatic steatosis (triglyceride accumulation in hepatocytes) to hepatic steatosis with inflammation (steatohepatitis), fibrosis, cirrhosis and hepatocellular carcinoma^1^. NAFLD is usually associated with obesity, type II diabetes, dyslipidemia, and some other metabolic syndromes^2,3^.

In patients with NAFLD, hepatic *de novo* lipogenesis (DNL) contributes most to the development of hepatic steatosis rather than high levels of plasma free fatty acids (FFAs), decreased hepatic β-oxidation or vLDL secretion^4^. DNL, synthesizing lipids by the use of nonfat materials, is accurately regulated by a group of enzymes in face to different nutritional conditions. For instances, high-carbohydrates-diet (HCD) feeding could enhance DNL of mouse liver, while fasting and high-fat-diet (HFD) feeding inhibit this process^5^. Moreover, some hormones are of crucial roles in regulating DNL, e.g. glucagon and catecholamine suppressing DNL while insulin and thyroid hormone stimulating the process^6–8^. Hepatic DNL is properly governed by complex gene regulatory networks. Transcription factors, such as sterol regulatory element-binding proteins 1c (SREBP-1c), liver X receptor (LXR), retinoid X receptor (RXR), and carbohydrate response element binding protein (ChREBP), regulate the expression of lipogenesis-associated enzymes^9–12^, including fatty acid synthase (FAS), and stearoyl-CoA desaturase 1 (SCD1), acetyl-CoA carboxylase (ACC) and ATP citrate lyase (ACLY)^12–14^. However, molecular mechanisms in regulating hepatic DNL still remain unclear. Therefore, molecular details of regulating DNL key enzymes are needed to be figured out for developing potential therapeutic approaches for NAFLD.

MicroRNAs (miRNAs), the small and noncoding RNA, regulate gene expression at posttranscriptional level through binding to mRNA of targeted genes and inhibiting translation or causing mRNA degradation, as well as promoting genes expression via binding to its promoter^15–18^. Given their critical role in lipid metabolism and insulin resistance, miRNAs now represent novel therapeutic target agents for human diabetes and metabolic disease^19,20^. MiR-27a was recently reported to repress lipid accumulation in rat hepatic stellate cell and human hepatoma cell by targeting *RXRα* ^21,22^ and impair adipocyte differentiation by targeting *PPARg* ^23^, indicating its potential role in lipid metabolism.

In our present study, we reported that overexpressed miR-27a attenuated DNL and lipid accumulation in both primary hepatocytes and mice livers by targeting 3′-UTR of *Fasn* and *Scd1* mRNAs. Ectopic expression of miR-27a reduced liver TG content by suppressing hepatic *Fasn* and *Scd1*, and thereby ameliorated NAFLD in HCD-fed and *ob/ob* mice. These results reveal the critical role of miR-27a-FAS/SCD1 axis in regulating lipid metabolism in liver and the development of NAFLD.

## Results

### miR-27a directly targets *Fasn* and *Scd1*

To identify miRNAs involved in the pathogenesis of NAFLD, we explored hepatic miRNAs with alterations in expression levels during the development of NAFLD. In consistent with previous studies, levels of miR-122 and miR-132 were upregulated in the fatty livers of obese mice caused by diets feeding (HFD or HCD) or genetic mutation (*ob/ob*) (Fig. S1). Strikingly, in comparison to control mice, hepatic miR-27a displayed about 4-fold increase in mice maintained on HFD for 12 weeks (Fig. S1a) and nearly 2-fold increase in *ob/ob* mice (Fig. S1c). Meanwhile, miR-27a exhibited an insignificant decrease in the livers of mice after feeding HCD for 8 weeks (Fig. S1b). To investigate molecular targets of miR-27a, we examined miR-27a-modulated signaling pathways by the using of miRNA target prediction algorithm Targetscan (http://www.targetscan.org). More than 900 candidate genes were predicted as targets of miR-27a and functional gene ontology enrichment analysis revealed most of these genes were involved in cytoskeleton remodeling and lipid metabolism signaling pathways, including lipogenesis-associated genes, such as *FAS*, *SCD1*, *PPARg* and *RXRa* (data not shown). As hepatic DNL is known to be stimulated by HCD feeding but inhibited by HFD feeding, we postulated that miR-27a was involved in the development of NAFLD via regulating DNL in liver.

**Figure 1:**
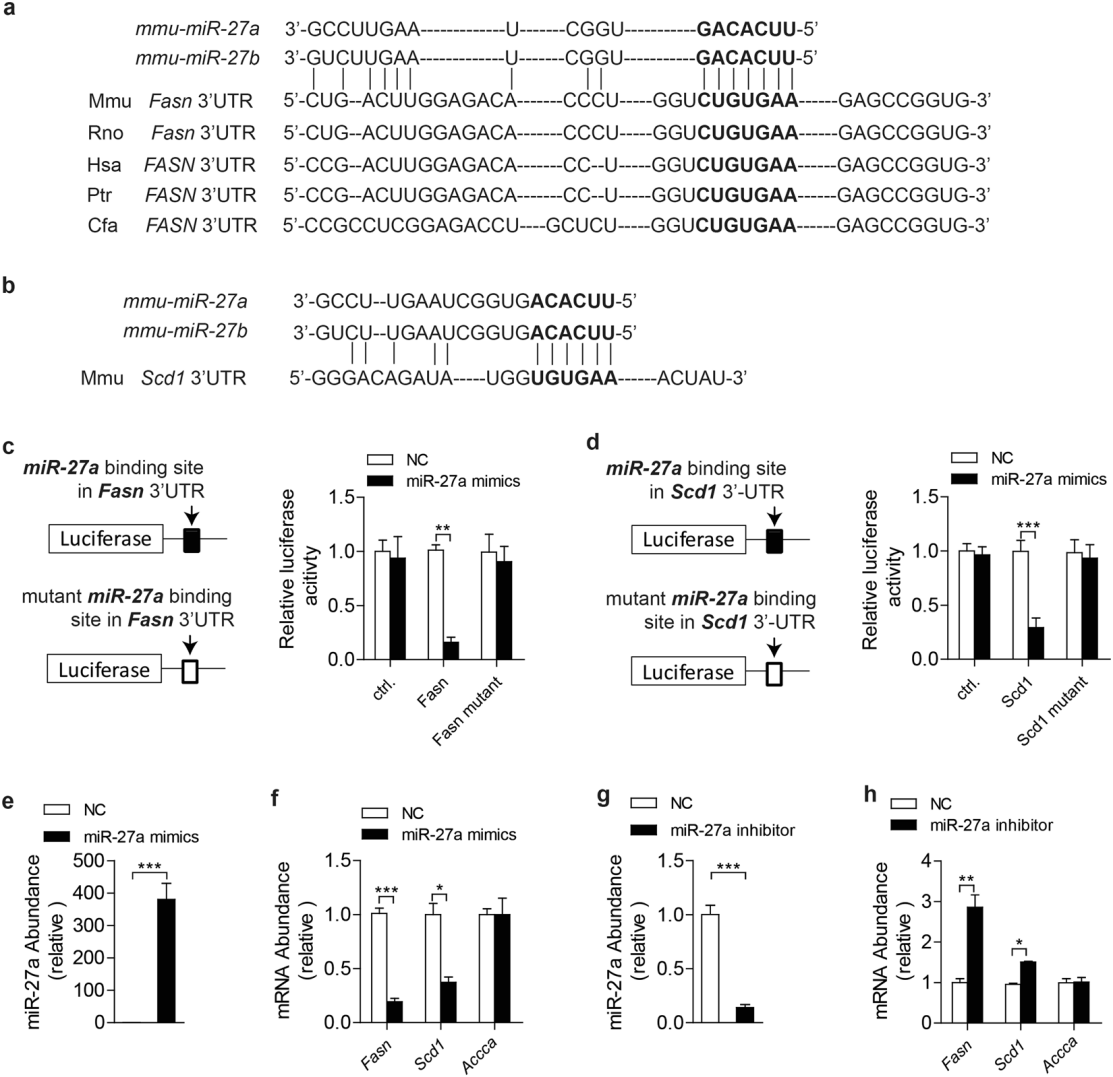
miR-27a targets 3′-UTR of mouse *Fasn* and *Scd1* mRNA. (**a**,**b**) Sequences alignment of mouse (mmu) miR-27a and miR-27b with the 3′-UTRs of *Fasn* mRNA of mouse (Mmu), human (Hsa), rat (Rno), chimpanzee (Ptr) and dog (Cfa) (**a**) and of *Scd1* mRNA of mouse (**b**). The seed regions of miR-27a and miR27b are indicated in bold. (**c**,**d**) Luciferase activity assays of luciferase reporter conjugated with 3′-UTR or 3′-UTR mutant (without predicted miR-27a binding site) of *Fasn* (**c**) and *Scd1* (**d**) in HEK293T cells. (**e**–**h**) Real-time PCR analysis of *miR-27a, Fasn, Scd1* and *Accca* in mouse primary hepatocytes transfected with miR-27a mimics (**e**,**f**) or miR-27a inhibitor (**g**,**h**). All data are shown as mean ± s.e.m. *p < 0.05, **p < 0.01, ***p < 0.001 by unpaired two-tailed student’s *t*-test.

Interestingly, *Fasn* and *Scd1* were found to be promising targets of miR-27a and miR-27b in sequence alignments (Fig. 1a,b). To determine miR-27a directly binding to 3′-UTR of *Fasn* and *Scd1*, renilla luciferase reporter plasmids were constructed with the insertion of predicted miR-27a binding sites inside of mouse *Fasn* and *Scd1* mRNA (Fig. 1c,d). Luciferase activities of wildtype 3′-UTRs reporter plasmids were dramatically downregulated after the addition of miR-27a mimics in HEK293T cells (Fig. 1c,d). However, mutation of predicted binding sites almost recovered the loss of luciferase activities mediated by excess miR-27a (Fig. 1c,d).

To determine whether miR-27a inhibiting hepatic expression of *Fasn* and *Scd1*, isolated mouse primary hepatocytes were transfected with miR-27a mimics to raise miR-27a levels (Fig. 1e) or with miR-27a inhibitors to reduce endogenous miR27a (Fig. 1g). Excess miR-27a attenuated expression levels of *Fasn* and *Scd1* but not *Accca* in primary hepatocytes (Fig. 1f). Moreover, the abrogation of endogenous miR-27a efficiently gave rise to mRNA levels of *Fasn* and *Scd1* rather than *Accca* (Fig. 1h). These data revealed that miR-27a regulated the stability of *Fasn* and *Scd1* mRNA by binding to their 3′-UTRs.

### miR-27a suppresses lipid accumulation in primary hepatocytes

**Figure 2:**
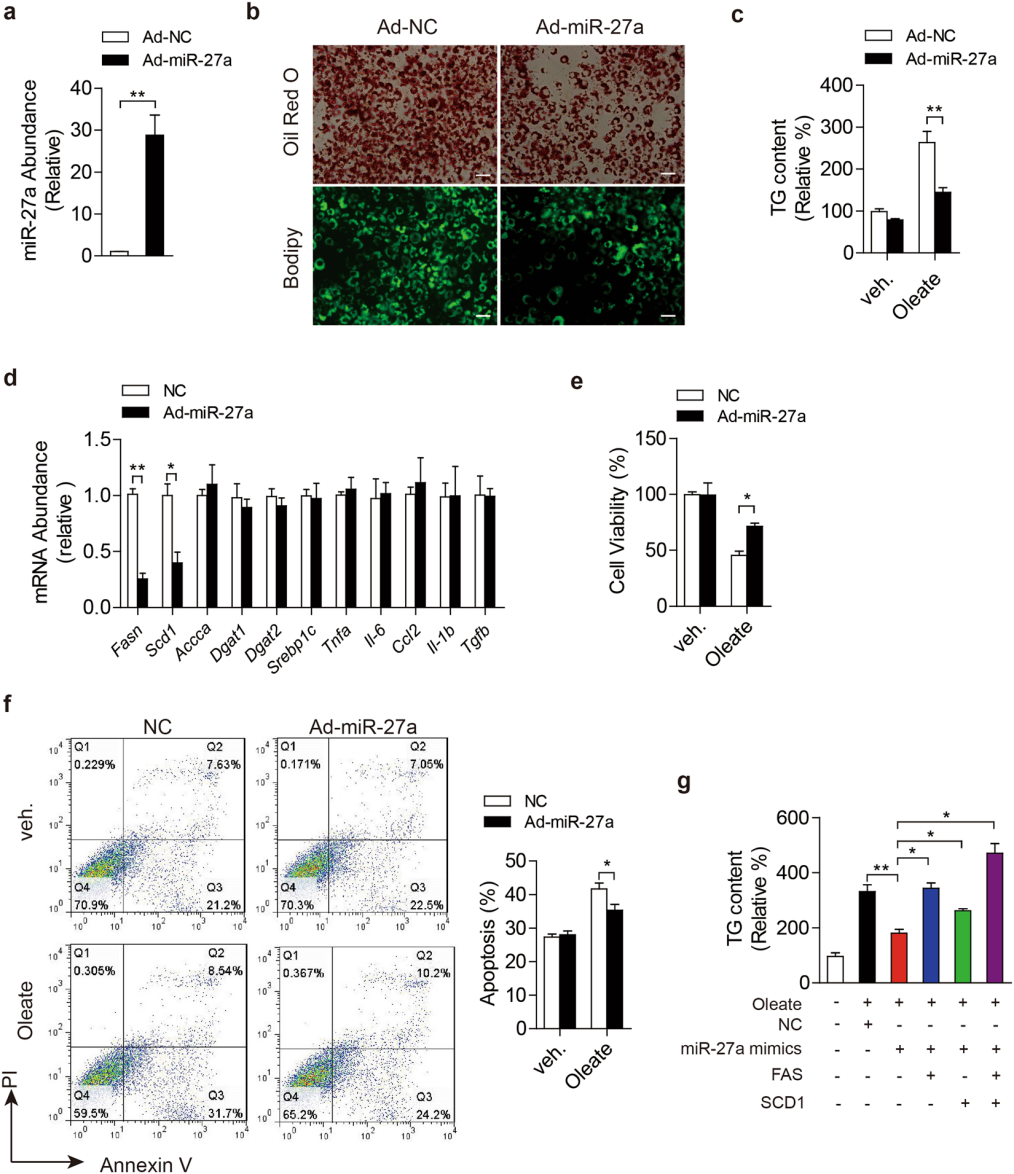
miR-27a reduces intracellular lipid accumulation in mouse primary hepatocytes. (**a**–**f**) Mouse primary hepatocytes were infected with adenovirus to enforce expression of scramble siRNA (Ad-NC) or miR-27a (Ad-miR-27a), and then treated with sodium oleate (1 mmol/l) for 24 hours. (**a**) miR-27a level; (**b**) Representative images of Oil-Red O staining (top) and Bodipy staining (lower). Scale bar is 100 μm. (**c**) Intracellular triglyceride (TG) content. (**d**) Real-time PCR analysis of genes involved in lipogenesis, inflammation and fibrosis. (**e**) Cell viability. (**f**) Flow cytometry analysis of cell apoptosis. (**g**) TG contents of sodium oleate-treated primary hepatocytes. The cells were firstly transfected with miR-27a mimics or combined with plasmids to overexpress *Fasn* and *Scd1*, and then treated with sodium oleate (1 mmol/l) for 24 hours. All data are shown as mean ± s.e.m. *p < 0.05, **p < 0.01 by unpaired two-tailed student’s *t*-test, one-way or two-way ANOVA.

To investigate physiological roles of miR-27a in hepatic lipid metabolism, mouse primary hepatocytes were infected by adenovirus to enforce miR-27a expression and then treated with sodium oleate to induce excess lipid accumulation. Impressively, excess miR-27a robustly impaired intracellular lipid accumulation and TG contents in oleate-treated primary hepatocytes (Fig. 2a–c). Adenovirus-mediated overexpression of miR-27a also led to the reduction of mRNA levels of *Fasn* and *Scd1* rather than other lipogenesis-associated genes, and didn’t affect the expression of cytokines involved in inflammation and fibrosis (Fig. 2d).

To detect whether excess miR-27a protect cells from lipotoxity, we examined cell viability by MTT assays and apoptosis using flow cytometry. Expectedly, overexpression of miR-27a strongly suppressed oleate-induced reduction of cell viability (Fig. 2e) and reduced cell apoptosis led by oleate treatment (Fig. 2f).

To explore the accurate extent of miR-27a excess which is efficient to repress TG accumulation, we transfected primary hepatocytes with serial volumes of miR-27a mimics from 0.1–100 pmol per well (24-well plate) and got different abundance of miR-27a in the cells (Fig. S2a). Impressively, ~10-fold increase of miR-27a acquired by transfection with 1 pmol/well was enough to get 20% reduction in TG accumulation induced by sodium oleate in primary hepatocytes (Fig. S2b).

To verify excess miR-27a reduce intracellular lipid accumulation via repressing *Fasn* and *Scd1*, we enforced expression of *Fasn* and *Scd1* in primary hepatocytes. Consistently, excess miR-27a alleviated oleate-initiated lipid accumulation of hepatoytes as well as excess miR-122 (Fig. 2g and S2c). Enforced expression of *Fasn* and *Scd1* abolished the inhibition effects of excess miR-27a on TG accumulation (Fig. 2g) but not those of miR-122 (Fig. S2c) in primary hepatocytes. Together, our results demonstrated that miR-27a suppressed oleate-induced hepatic lipid accumulation via suppressing *Fasn* and *Scd1* in primary hepatocytes.

### Ectopic expression of miR-27a alleviated HCD-induced NAFLD via repressing lipogenesis in mice liver

**Figure 3:**
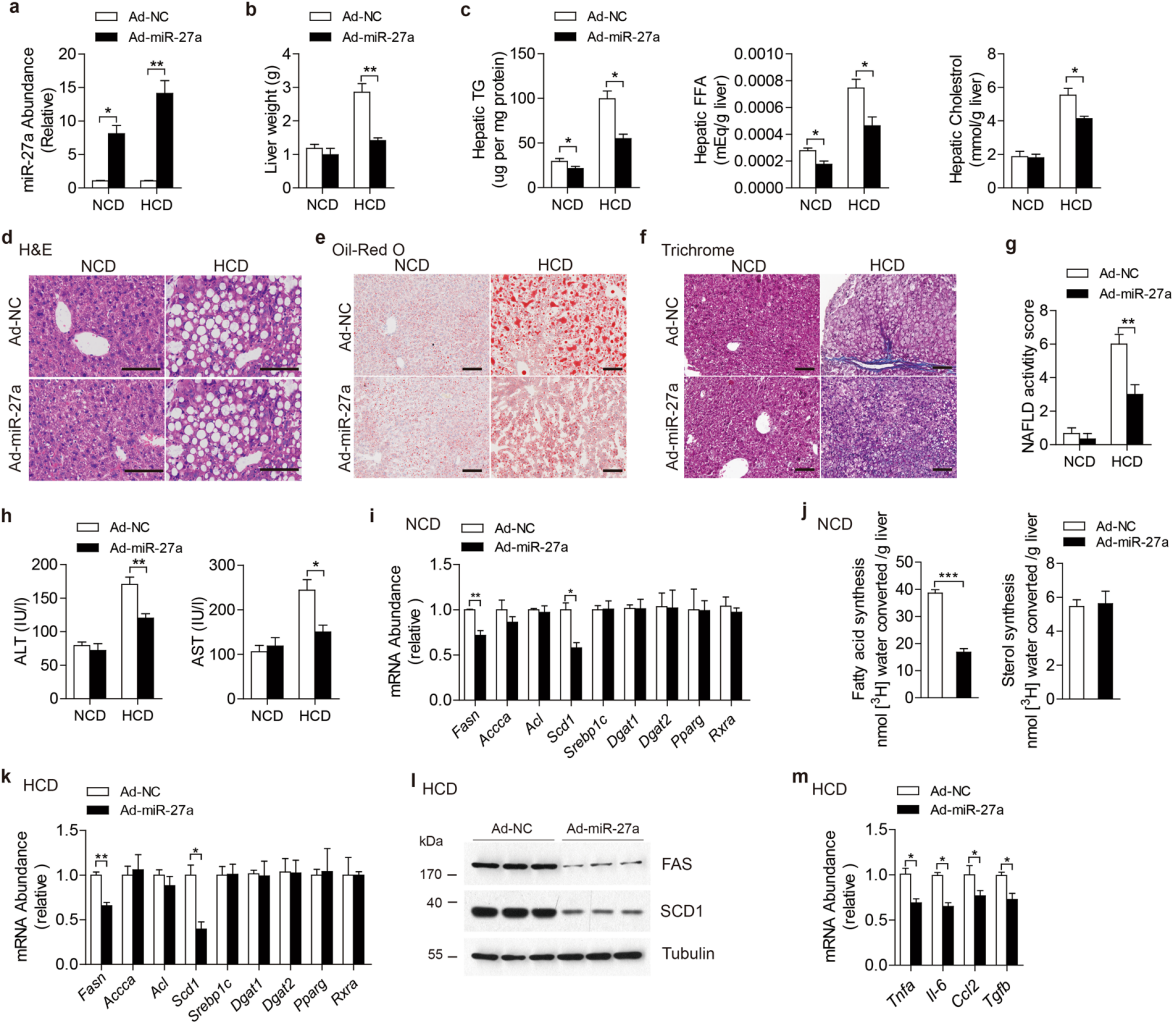
Ectopic expression of miR-27a attenuates hepatic lipid accumulation and improves HCD-induced NAFLD via repressing hepatic lipogenesis in mice. C57BJ/6 L fed on normal chow diet (NCD, n = 12 per group) or high carbohydrate diet (HCD, n = 10 per group) for 8 weeks and then administered with indicated adenovirus. (**a**) Real-time PCR analysis of miR-27a abundance in livers. (**b**) Liver weight. (**c**) Hepatic contents of TG, free fat acids (FFA) and cholesterol. (**d**–**f**) Representative images of H&E staining (**d**), Oil-Red O staining (**e**) and Masson’s trichrome staining (**f**) of liver slides. Scale bar is 100 μm. (**g**) NAFLD activity score. (**h**) Serum ALT and AST levels. (**i**) Real-time PCR analysis of genes involved in hepatic lipogenesis of NCD-fed mice. (**j**) Synthesis of fatty acids and sterol were analyzed in livers of NCD-fed mice. (**k**–**m**) Real-time PCR analysis of lipogenesis-associated genes (**k**), immunoblotting analysis for indicated proteins (**l**) and real-time PCR analysis of proinflammatory and fibrogenic cytokines (**m**) in livers of HCD-fed mice. All data are shown as mean ± s.e.m. *p < 0.05, **p < 0.01, ***p < 0.001by unpaired two-tailed student’s *t*-test or two-way ANOVA.

To explore the physiological role of miR-27a *in vivo*, C57BL/6 J mice were injected with adenovirus via tail vein to enforce its expression in livers. As expected, miR-27a levels were found to increase about 10 times in livers (Fig. 3a) but displayed no difference in other major metabolic organs, including skeletal muscle and adipose tissue (Fig. S4a,b) of mice administered by ad-miR-27a relative to that of ad-NC. Significant alterations of body weight and food intake weren’t detected in mice after ad-miR-27a administration (Fig. S3a,b). Impressively, with similar liver weight (Fig. 3b), ad-miR-27a-injected mice exhibited significant reduction of hepatic TG contents relative to control mice (Fig. 3c). Administration of ad-miR-27a also displayed reduced contents of free fatty acid (FFA) but equal cholesterol contents in livers (Fig. 3c).

Subsequently, mice were maintained on HCD for 8 weeks to induce NALFD and then administered with ad-miR-27a to increase miR-27a expression levels in livers (Fig. 3a). Relative to control mice, we didn’t detect any changes of miR-27a levels in skeletal muscle or adipose tissue in the mice administered with ad-miR-27a (Fig. S4a,b). With similar body weight and food intake as control mice (Fig. S3a,b), ad-miR-27a-injected mice exhibited reduced liver weight (Fig. 3b) and lower hepatic lipids contents, including TG, FFA and cholesterol (Fig. 3c). Histology analysis also demonstrated decreased lipid accumulation in the livers of mice with excess hepatic miR-27a (Fig. 3d,e), indicating alleviated hepatic steatosis. Moreover, results of trichrome staining of miR-27a-overexpressed livers showed robust reduction of fibrosis and lower NAFLD activity score (Fig. 3f,g), reflecting improved NAFLD development. Further studies also revealed overexpression of hepatic miR-27a efficiently repressed HCD-initiated liver damage (Fig. 3h), hyperglycemia and hyperinsulinemia (Fig. S3d), indicating improved systemic glucose homeostasis and insulin sensitivity.

Hepatic TG content is highly dynamic and contributed by various processes, including TG synthesis, β-oxidation, FFA uptake and vLDL secretion^24–26^. Therefore, we analyzed expression levels of genes involved in these processes. In consistent to the results of *ex vivo* study, in the livers of NCD-fed mice, upregulated miR-27a resulted in dramatic reduction of *Fasn* and *Scd1* mRNA (Fig. 3i), but didn’t alter expression levels of other lipogenic-associated genes, including *Accca, Dgat1, Dgat2, Acl, Srebp1c, Pparg* and *Rxra* (Fig. 3i). In comparison with control mice, ad-miR-27a-treated mice didn’t show any significant changes of mRNA levels of genes involved in hepatic β–oxidation, FFA intake, vLDL secretion and gluconeogenesis, as well as hepatic inflammation and fibrosis in livers under NCD feeding (Fig. S3f). To examine DNL in liver, ^3^H-water was injected into mice as a tracer for new synthesized lipids. Impressively, livers of ad-miR-27a-administered mice displayed a substantial reduction in the synthesis of fatty acids but not sterols relative to control livers (Fig. 3j), indicating impaired lipogenesis in miR-27a-overexpressed livers. In HCD-fed mice, excess hepatic miR-27a led to a much lower levels of FAS and SCD1 in both mRNA and protein in liver (Fig. 3k,l), but not in skeletal muscle and adipose tissue (Fig. S4c,d). The miR-27a-overexpressed livers exhibited unaltered expression of other lipogenic-associated genes (Fig. 3k), as well as genes involved in hepatic β–oxidation, FFA intake, vLDL secretion and gluconeogenesis (Fig. S3g), but much lower expression of inflammatory and fibrogenic cytokines (Fig. 3m), indicating alleviated hepatitis and fibrosis.

### Ectopic expression of miR-27a improved NALFD in obesity mice

**Figure 4:**
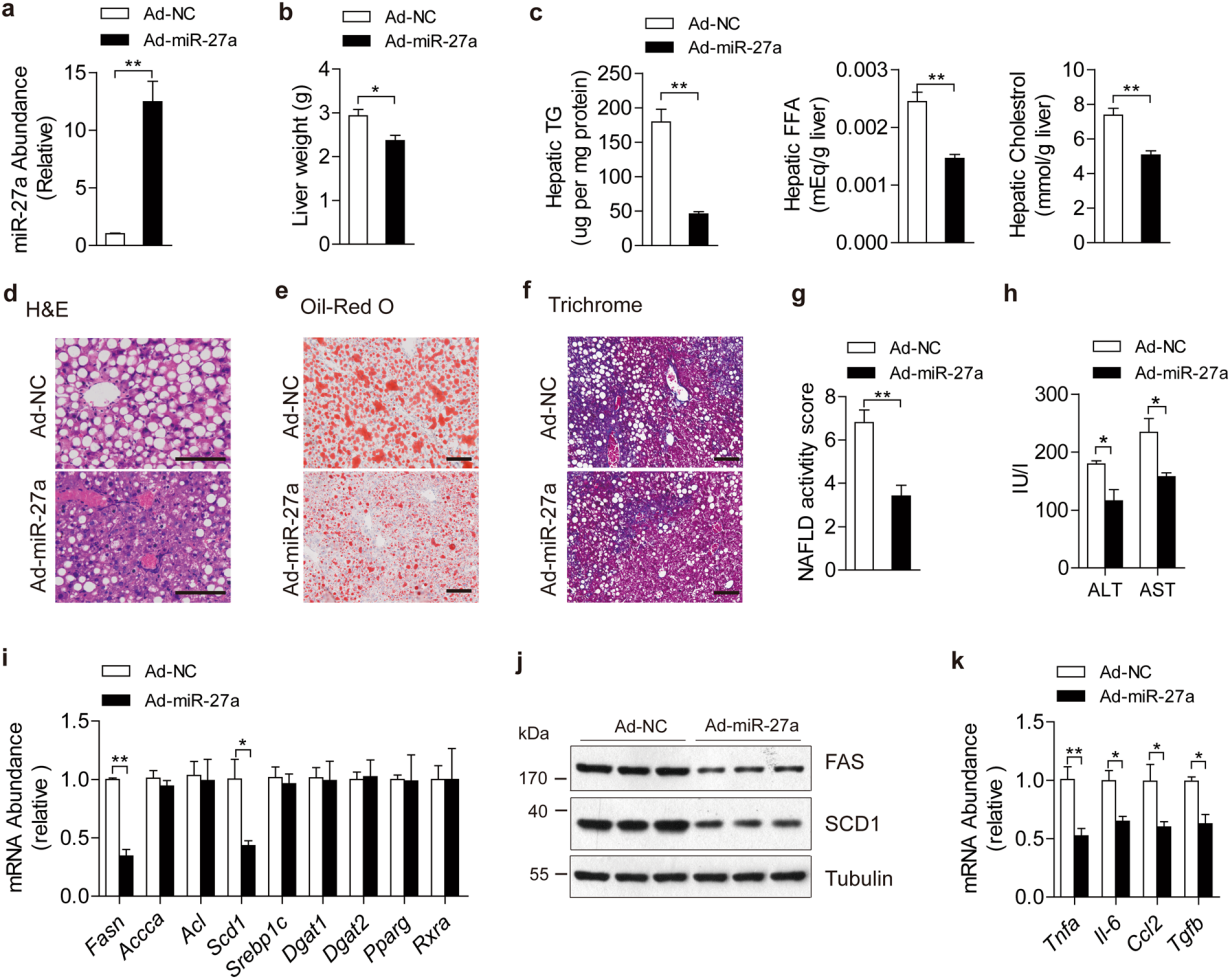
Ectopic expression of miR-27a alleviates hepatosteatosis and NAFLD in *ob/ob* mice. 16-week-old *ob/ob* mice were administered with Ad-NC and Ad-miR-27a (n = 8 per group). (**a**) Real-time PCR analysis of miR-27a abundance in livers. (**b**) Liver weight. (**c**) Hepatic contents of TG, FFA and cholesterol. (**d**–**f**) Representative images of H&E staining (**d**), Oil-Red O staining (**e**) and trichrome staining (**f**) of liver slides. Scale bar is 100 μm. (**g**) NAFLD activity score. (**h**) Serum ALT and AST levels. (**i**–**k**) Real-time PCR analysis of lipogenesis-associated genes (**i**), immunoblotting analysis for indicated proteins (**j**) and real-time analysis of proinflammatory and fibrogenic cytokines (**k**) in livers. All data are shown as mean ± s.e.m. *p < 0.05, **p < 0.01 by unpaired two-tailed student’s *t*-test.

To further determine the roles of miR-27a in obesity-induced NAFLD, *ob/ob* mice were administered with ad-miR-27a to enforce the expression of miR-27a in livers instead of skeletal muscle and adipose tissue (Figs 4a and S6a). Relative to control mice, excess hepatic miR-27a in *ob/ob* mice resulted in marked reductions in liver weight (Fig. 4b) and hepatic contents of TG, FFA and cholesterol (Fig. 4c), impaired hepatosteatosis (Fig. 4d,e), improved NAFLD (Fig. 4f,g) and liver damage (Fig. 4h), but the same levels body weight and food intake (Fig. S5a,b). Mechanistically, upregulated hepatic miR-27a also attenuated mRNA and protein levels of FAS and SCD1 in livers (Fig. 4I,j) but not in skeletal muscle or adipose tissue (Fig. S6b,c). Ectopic expression of miR-27a in liver didn’t disturb mRNA levels of other lipogenesis-associated genes (Fig. 4i) or genes involved in β–oxidation, FFA intake and vLDL secretion (Fig. S5f). Notably, ad-miR-27a-administered *ob/ob* mice also displayed reduced expression of proinflammatory and fibrogenic genes in liver (Fig. 4k), decreased plasma TG and improved hyperglycemia and hyperinsulinemia (Fig. S5c,d). Taken together, these data revealed that miR-27a acts as a crucial repressor in hepatic lipogenesis and suppressed the development of obesity-induced NFALD.

## Discussion

A growing number of studies implicate miRNAs as crucial mediators in regulating lipid homeostasis. MiR-122 is the first miRNA which was discovered to be involved in the regulation of lipid synthesis, catabolism and secretion of liver^27,28^. Subsequent studies in mice demonstrated miR-34a contributing to hepatic steatosis via repressing sirtuin 1 (*Sirt1*)^29^, miR-132 and miR-30 regulating hepatic lipid synthesis and lipoprotein secretion^30,31^, and miR-29 involved in lipogenic programs of liver^32^. Vickers *et al*. reported that miR-27b is a regulatory hub in hepatic lipid metabolic networks^33^. In our present study, miR-27a was revealed to act as a critical repressor in regulating hepatic DNL and obesity-initiated NAFLD progression. MiR-27a targeted 3′-UTRs of mouse *Fasn* and *Scd1* mRNAs. Overexpressed miR-27a reduced lipid accumulation in primary hepatocytes and mice liver by repressing expression levels of *Fasn* and *Scd1*. Ectopic expression of miR-27a in liver attenuated the development of NAFLD induced by HCD feeding and genetic obesity through impairing lipogenic programs.

Previous study indicated miR-27a is involving in lipid metabolism and inhibits lipid droplets formation in rat hepatic stellate cells^22^. Shirasaki *et al*. also demonstrated miR-27a could impair oleic acid-induced lipid accumulation and regulate lipid metabolism in human hepatoma cells through targeting *RXRa* and *ABCA1*, and then inhibit Hepatitis C virus replication^21^. In our data, overexpression of hepatic miR-27a dramatically blocked sodium oleate-induced lipid accumulation in mouse primary hepatocytes (Fig. 2) and reduced hepatic TG content by impairing DNL of liver (Fig. 3). Although RXRα is a critical mediator in hepatic DNL process, we didn’t find overexpressed hepatic miR-27a affected mRNA levels of *Rxra* in liver, skeletal muscle or adipose tissue of NCD-fed or HCD-fed mice or *ob/ob* mice (Figs 2–4 and S4 and S6), which is inconsistent with previous reports^21,22^. These differences between our present work and previous studies may be ascribed to different types of cells used in experiments. Our experiments were all conducted in mouse primary hepatocytes and livers, of which metabolic processed and genes expression patterns are not identical with that of human hepatoma cells.

MiR-27a was identified as a negative regulator of adipocyte differentiation by targeting *PPARg* ^23^, which is also a critical mediator in hepatic DNL. However, we didn’t find overexpressed hepatic miR-27a displayed any influence on the expression of *Pparg* in primary hepatocytes, or liver, skeletal muscle and adipose tissue of NCD-fed or HFD-fed mice or *ob/ob* mice (Figs 2–4 and S5 and S7). These results exclude the possibility that miR-27a exerts its functions on hepatic lipid metabolism via targeting *Pparg*.

To compare miR-27a with other obesity-modified miRNAs, expression levels of hepatic miR-122 and miR-132 were also analyzed and found to be dramatically upregulated in livers of HFD-fed and *ob/ob* mice (Fig. S1) as described in previous reports^27,30^. Interestingly, in these fatty livers, miR-27a displayed a similar change as miR-122 and miR-132 did. But in livers of HCD-feeding mice, which hepatic lipogenesis is robustly promoted, miR-27a exhibited an insignificant reduction while miR-122 and miR-132 were dramatically upregulated (Fig. S2b). Furthermore, ectopic expression of miR-27a or miR-122 could block sodium oleate-induced TG accumulation in primary hepatocytes, respectively (Figs 1g and S2b). Surprisingly, enforced expression of *Fasn* and *Scd1* could abolish the inhibition effects of excess miR-27a on TG accumulation (Fig. 1g) but not affect those of miR-122 (Fig. S2b) in primary hepatocytes. These results suggest the functions and underlying molecular mechanisms of miR-27a might be different from other obesity-modified miRNAs (e.g. miR-122 and miR-132) in various contexts of fatty livers.

We firstly reported miR-27a directly targeted 3′-UTR of mouse *Fasn* and *Scd1*, and regulated their expression in mouse primary hepatocytes (Fig. 1) and livers (Fig. 2). Our results revealed that miR-27a exerted its functions in hepatic lipid metabolism via regulating DNL rather than disturbing β-oxidation, FFA uptake and vLDL secretion (Fig. 3), indicating its important role in the pathogenesis of NAFLD. DNL is considered as the main reason for fatty liver induced by HCD feeding rather than HFD feeding^34,35^. The increase of hepatic miR-27a mediated by adenovirus sufficiently inhibited hepatic TG, lipid droplets formation and hepatic steatosis in livers of mice maintained on HCD (Fig. 3). Furthermore, ectopic expression of miR-27a repressed the expression of *Fasn* and *Scd1* rather than other genes involved in hepatic lipid metabolism (Fig. 3). We also investigated miR-27a’s effects on obesity-induced NAFLD by applying ad-miR-27a to *ob/ob* mice. Similarly, enhanced expression of hepatic miR-27a alleviated the development of NAFLD in obese mice through inhibiting *Fasn* and *Scd1* expression and hepatic lipogenesis (Fig. 4). Our results reveal miR-27a acts as a negative regulator in hepatic DNL and suppresses the development of NAFLD induced by HCD and genetic obesity.

In determining the accurate extent of miR-27a excess which is efficient to repress TG accumulation, we found that ~10-fold increase of miR-27a could lead to a 20% reduction of TG accumulation induced by sodium oleate in primary hepatocytes (Fig. S3b). In our studies of *in vivo*, we found that a ~10-fold in NCD-feeding mice, or a ~15-fold in HCD-feeding and *ob/ob* mice (Figs 3a and 4a) increase of hepatic miR-27a were efficient enough to repress hepatic Fas and Scd1 expression and therefore alleviate NAFLD. Further studies are indeed needed to explore an exact extent of miR-27a expression which is required to improve obese-initiated NAFLD.

Further studies revealed that except of liver, adenovirus administration didn’t lead to the changes of miR-27a expression in other main metabolic tissues (including skeletal muscle and adipose tissue) (Figs S4 and S6), indicating miR-27a of skeletal muscle or adipose tissue doesn’t contribute to the phenotypes in our present study.

MiR-27a excess was reported to act as an oncogene in the development of gastric and nasopharyngeal cancers^36,37^. However, there are still no published studies implicating that hepatic miR-27a promotes liver cancer development. Impressively, miR-27a was revealed to be downregulated in human HCC samples and plays as a tumor suppressor in tumor metastasis and vasculogenic mimicry via targeting *Twist-1* in HCC^38^. With the fact that obesity-initiated NAFLD markedly increases the risk of HCC, the benefic effects of miR-27a on fatty liver and NAFLD in our studies suggest that excess miR-27a could suppress obesity-induced HCC pathogenesis.

In summary, our study firstly reveals a novel and critical role of miR-27a in regulating hepatic DNL and NAFLD development via targeting *Fasn* and *Scd1*. These findings provide new insights for understanding obesity-associated NAFLD progression and also novel promising therapeutic target for NAFLD.

## Methods

### Animals and treatment

Male C57BL/6J mice of 8-week old were purchased from SILAC Animal Research Center of Shanghai (Shanghai, China), and then fed on normal chow diet (NC), high carbohydrate diet (HCD) rich in fructose and sucrose (72.2% carbohydrates, 1% fat, and 26.8% protein by energy, Research Diet Inc., USA) or high fat diet (20% carbohydrates, 60% fat, and 20% protein by energy, Research Diet Inc., USA). Genetically diabetic leptin deficient (*ob/ob*) mice were purchased from Nanjing University. Mice were maintained on a 12 h light/dark cycle at 22 °C and provided with free access to food and tap water.

For overexpression of miR-27a, male C57BL/6J mice at the age of 10weeks, *ob/ob* mice at the age of 16 weeks or 14-week-old C57BL/6J mice after maintained on HCD for 8 weeks were injected intravenously through the tail vein with adenovirus encoding scramble RNA (Ad-NC) or miR-27a (Ad-miR-27a) with PFU at 5 × 10^9^ in 1 mL PBS. Mice were sacrificed 2~3 weeks later after adenovirus injection. Mice were starved for 6 hours for fasting glucose determination. All experiments in this study were performed in accordance with protocols approved by the Institutional Animal Care and Use Committee of Fudan University.

### Cell culture

Primary hepatocytes were isolated from livers of male C57BL/6J mice at the age of 10 weeks as previous description^14,39^. Prepared primary hepatocytes were maintained in Dulbecco’s modified Eagle’s medium (DMEM) in addition with 25 mmol/l glucose (Gibco, Invitrogen), 10% FBS (Gibco, Invitrogen), 50 mg/ml penicillin and streptomycin at 37 °C, and 5% CO_2_–95% air. MiRNA double-stranded mimics for miR-27a and miR-122, and inhibitors for miR-27a were obtained from Qiagen. Primary hepatocytes were transfected with miR-27a mimics (10 pmol/ml), inhibitors (50 pmol/ml), miR-122 mimics (5 pmol/ml) or negative control (10 pmol/ml) using HiPerFect (Qiagen) as per manufacturer’s instructions and following experiments were performed 48 h after transfection.

For induction of intracellular lipid accumulation, primary hepatocytes were cultured in a medium with the addition of 1 mmol/l sodium oleate (Sigma) for 24 hours and then the cells were harvested for further analysis. Oil-Red O and Bodipy staining of cells were conducted as previously described. Cell viability assays were conducted by the using of MTT (Sigma) and apoptosis were determined by Annexin V and PI staining (Biyuntian Biotech) according to the manufacturers’ instructions.

### DNA constructs and mutagenesis

mRNA of mouse *Fasn* and *Scd1* containing potential miR-27a binding sites were cloned into pMiR-GLO vector (Invitrogene). Mutated reporter plasmids were constructed through deleting potential miR-27a binding sites under the instruction of gene mutation kit (Beijing Dingguo).

### Luciferase assay

Plasmids containing either wild-type or mutated 3′-UTR sequence were transfected into HEK293T cells using Lipofectamine™ 2000 Transfection Reagent (Invitrogen) and Opti-MEM (Invitrogen) as instruction, and renilla plasmid was co-transfected as the transfection control. The cells were also co-transfected with either the negative control or the mimic (10 pmol/ml) and incubated for 24 h, and then lysed using 1 × Passive Lysis Buffer (Promega Dual Luciferase Assay Kit, Promega). The lysis was applied to luciferase activity test using a luminometer (Orion II Luminometer) according to the manufacturer’s instructions. Firefly luciferase values were normalized to those of Renilla luciferase.

### Generation and administration of recombinant adenoviruses

The recombinant adenoviruses expressing miR-27a or scramble RNA were generated by the use of AdEasy^TM^ Vector System (Invitrogene). Viruses were collected and purified as described^14^. Viruses were diluted in PBS and administered at a dose of 1 × 10^7^ pfu/well in 12-well plate or through tail vein injection in a dose of 1 × 10^9^ pfu/mice.

### *In vivo* lipogenesis assay

Hepatic *de novo* lipogenesis *in vivo* was measured as previously described^40^ with some modifications. In brief, mice were i.p. injected with 0.5 ml of 0.15 mol/l NaCl containing 0.2 mCi of [^3^H]-water/100 g body weight. About 0.7 g liver sample was excised from one mouse one hour later, and heated at 90 °C for 5 hours in a mixture of 1.5 ml of 4 mol/l KOH and 1.5 ml of 95% ethanol, and then mixed with 4 ml of hexane. After centrifugation, we collected the organic phase, and dried for [^3^H]-sterol assay. The aqueous phase (3 ml) was acidified with 0.75 ml of 10 mol/l H2SO4 before mixing with 4 ml hexane, and subjected to centrifugation. Then the organic phase was washed with 3 ml of distilled water, and dried for the determination of [^3^H]-labeled fatty acids and sterols.

### RNA isolation and real-time PCR

Total RNA was prepared from cells or frozen tissues using TRIzol (Invitrogen) reagent. The 2 μg total RNA was reversely transcribed with random primer (Life Technology) and M-MLV Reverse Transcriptase (Invitrogen).

For miRNA, total RNA were added polyA tail catalyzed by *E. coli* polyA polymerase (NEB). Thereby, 1 μg of the tailed RNA was reverse transcribed using miR dTRT primer (5′-cgactcgatccagtctcagggtccgaggtattcgatcgagtcgcacttttttttttttv-3′). The abundance of miR-27a, miR-122 and miR-132 were determined by the using of syberGreen Real-time PCR mixture (Roche) with specific primers obtained from Exiqon and U6 was used as internal control.

mRNA abundance was also determined by the using of syberGreen Real-time PCR mixture (Roche) and mouse 18 s rRNA was used as internal control. The sequences of primers mainly used in this study showed as following.

*18s rRNA*: sense 5′-AGGGGAGAGCGGGTAAGAG-3′, antisense5′-GGACAGGACTAGGCGGAACAACA-3′;

*Fasn*: sense 5′-GGCTCTATGGATTACCCAAGC-3′, antisense 5′-CCAGTGTTCGTTCCTCGGA-3′;

*Scd1*: sense 5′-AGATCTCCAGTTCTTACACGACCAC-3′, antisense 5′-GACGGATGTCTTCTTCCAGGTG-3′;

*Ppara*: sense 5′-AGAGCCCCATCTGTCCTCTC-3′, antisense 5′-ACTGGTAGTCTGCAAAACCAAA-3′;

*Pgc1a*: sense 5′-TATGGAGTGACATAGAGTGTGCT-3′, antisense 5′-CCACTTCAATCCACCCAGAAAG-3′;

*Acc1*: sense 5′-GATGAACCATCTCCGTTGGC-3′, antisense 5′-CCCAATTATGAATCGGGAGTGC-3′;

*Cpt1a*: sense 5′-CTCCGCCTGAGCCATGAAG-3′, antisense 5′-CACCAGTGATGATGCCATTCT-3′;

*Cd36*: sense 5′-GAACCACTGCTTTCAAAAACTGG-3′, antisense 5′-TGCTGTTCTTTGCCACGTCA-3′;

*ApoB*: sense 5′-GCTCAACTCAGGTTACCGTGA-3′, antisense 5′-AGAGGGCTGACACTTGTACTG-3′;

*Srebp1c*: sense 5′-GGAGCCATGGATTGCACATT-3′, antisense 5′-GGCCCGGGAAGTCACTGT-3′;

*Apoe*: sense 5′-CTCCCAAGTCACACAAGAACTG-3′, antisense 5′-CCAGCTCCTTTTTGTAAGCCTTT-3′;

*Abca1*: sense 5′-AAAACCGCAGACATCCTTCAG-3′, antisense 5′-CATACCGAAACTCGTTCACCC-3′;

*Pparg*: sense 5′-TCGCTGATGCACTGCCTATG-3′, antisense 5′-GAGAGGTCCACAGAGCTGATT-3′;

*Rxra*: sense 5′-ATGGACACCAAACATTTCCTGC-3′, antisense 5′-CCAGTGGAGAGCCGATTCC-3′;

*Acly*: sense 5′-ACCCTTTCACTGGGGATCACA-3′, antisense 5′-GACAGGGATCAGGATTTCCTTG-3′;

*ApocIII*: sense 5′-TACAGGGCTACATGGAACAAGC-3′, antisense 5′-CAGGGATCTGAAGTGATTGTCC-3′;

*Ces1*: sense 5′-CTGCTTGCTTGAGTCTGGGAC-3′, antisense 5′-TTGGTAGCACAAAGGAGGGTA-3′;

*Dgat1*: sense 5′-TCCGTCCAGGGTGGTAGTG-3′, antisense 5′-TGAACAAAGAATCTTGCAGACGA-3′;

*Dgat2*: sense 5′-GCGCTACTTCCGAGACTACTT-3′, antisense 5′-GGGCCTTATGCCAGGAAACT-3′;

*Pepck*: sense 5′-AAGCATTCAACGCCAGGTTC-3′, antisense 5′-GGGCGAGTCTGTCAGTTCAAT-3′;

*G6pase*: sense 5′-CGACTCGCTATCTCCAAGTGA-3′, antisense 5′-GTTGAACCAGTCTCCGACCA-3′;

*Tnfa*: sense 5′-GACGTGGAACTGGCAGAAGAG-3′, antisense 5′-ACCGCCTGGAGTTCTGGAA-3′;

*Il-6*: sense 5′-CCACGGCCTTCCCTACTTC-3′, antisense 5′-TTGGGAGTGGTATCCTCTGTGA-3′;

*Ccl2*: sense 5′-TTAAAAACCTGGATCGGAACCAA-3′, antisense 5′-GCATTAGCTTCAGATTTACGGGT-3′;

*Tgfb*: sense 5′-CCACCTGCAAGACCATCGAC-3′, antisense 5′-CTGGCGAGCCTTAGTTTGGAC-3′.

### Immunoblotting analysis

Immunoblotting was performed as previous describe^39^. In brief, mouse liver tissue and cells were homogenized by RIPA lysis buffer (150 mM Tris-HCl, 50 mM NaCl, 1% NP-40, 0.1% tween-20), and centrifuged at 15,000 g for 15 min. Then, the supernatant was mixed with loading buffer [125 mM Tris hydrochloride (pH 6.8), 10% mercaptoethanol (vol/vol), 4% SDS (wt/vol), 20% glycerol (vol/vol), and 0.002% bromophenol blue] and then was heated at 100 °C for 10 min. Supernatants were subjected to 10% SDS-PAGE gels. The primary antibodies were incubated overnight at 4 °C and visualized by ECL Plus (Thermo). Band intensities were measured by Image. J software and normalized to Tubulin.

### Triglyceride, free fat acids and cholesterol test

The determination of TG, FFA and cholesterol were performed according to previous description^41^. Prepared primary hepatocytes were transfected with either miR-27a mimic or ad-miR-27a, and then were scraped and centrifuged after incubation for 48 h. Livers tissues were homogenized with PBS. Cellular and hepatic lipids were extracted with the mixture of chloroform and methanol (2:1) as described previously^42^. Hepatic and plasma TG, cholesterol and FFA were measured by colorimetric assays (Sigma and Wako) according to the manufacturers’ instructions, respectively.

### Histological analysis, Oil-Red O staining and Masson’s trichrome staining of livers

For Oil-Red O staining, liver samples were embedded in Tissue-Tek OCT embedding compound, and frozen on dry ice. 8 μm-thick frozen sections were cut were stained with an Oil-red O (Sigma). For H&E staining and Masson’s trichrome staining, liver tissues were fixed by paraformaldehyde and embedded in paraffin. 8 μm-thick sections of livers were cut and stained with hematoxylin and eosin for H&E staining, or trichrome staining for collagen by the using of Trichrome Stain Kit (Sigma) according to manufacturer’s instruction. Images were taken with Zeiss Axioplan 2 Upright Microscope.

TUNEL staining to detect death cells were performed by the using of DeadEnd Fluorometric TUNEL System (Promega) according to manufacturer’s instruction.

### Statistical analysis

All data are expressed as the mean ± s.e.m. of at least three independent experiments. Significant differences were analyzed according to indicated statistical methods using GraphPad Software. *p* < 0.05 was considered statistically significant.

### Data Availability Statement

The datasets used and/or analyzed during the current study available from the corresponding author on reasonable request.

## Electronic supplementary material

Supplementary Information
