# Supplementary material for: MicroRNA-27a regulates hepatic lipid metabolism and alleviates NAFLD via repressing FAS and SCD1
Source: Sci Rep. 2017 Nov 3;7:14493. doi: 10.1038/s41598-017-15141-x (PMC5670231; doi:10.1038/s41598-017-15141-x)
Supplement: Supplementary file 1 — Supplementary Information [file 41598_2017_15141_MOESM1_ESM.doc]

**Supplementary Information**

**MicroRNA-27a regulates hepatic lipid metabolism and alleviates NAFLD via repressing FAS and SCD1**

Meiyuan Zhang^1^, Weilan Sun^1^, Minghao Zhou^1^, Yan Tang^1,*^

^1^Emergency Intensive Care Unit, Qingpu Branch of Zhongshan Hospital, Fudan University, Shanghai201700, China.

^*^ Corresponding Author.

Emergency Intensive Care Unit, Qingpu Branch of Zhongshan Hospital, Fudan University, Shanghai201700, China.

E-mail: yantang_zsh@163.com.


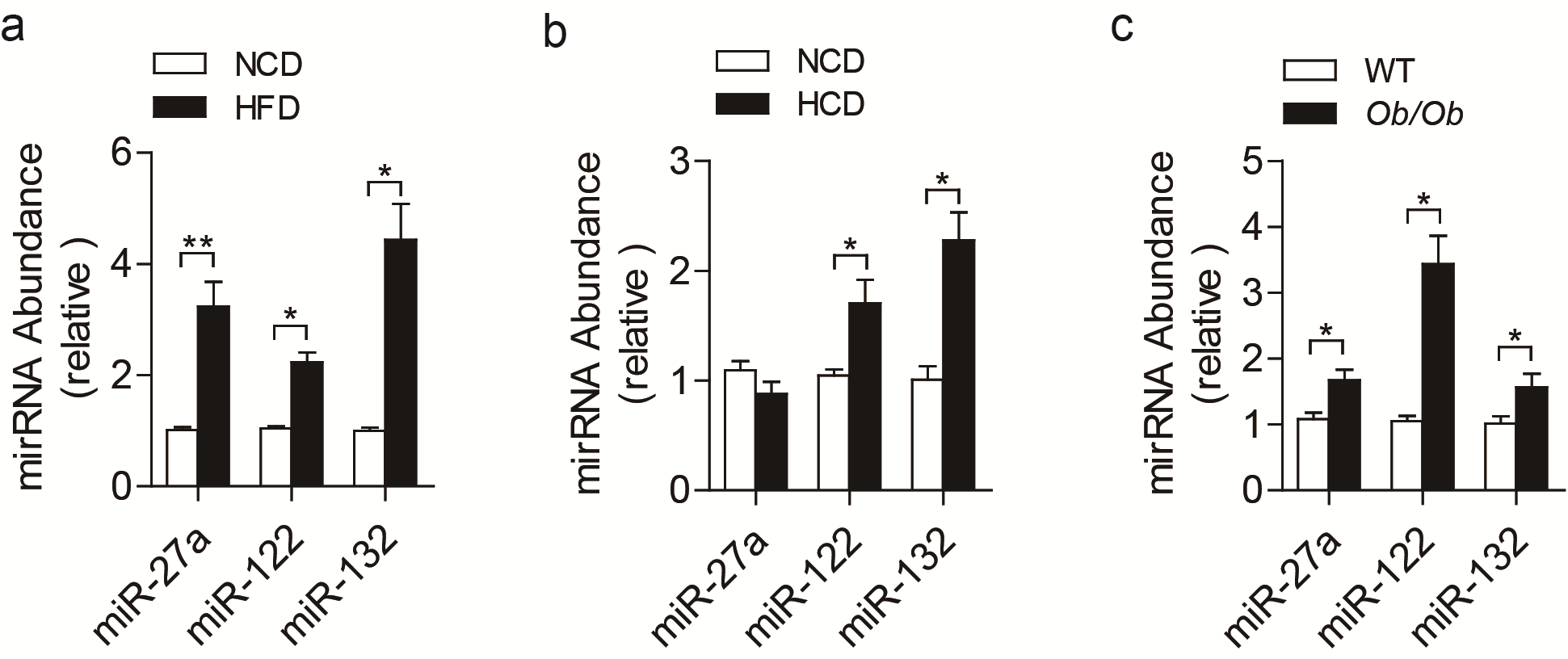


**Supplementary Figure 1. Expression levels of miR-27a increase in livers of obese mice induced by HFD or genetic loss of leptin.** Real-time PCR analysis of RNA levels of miR-27a, miR-122 and miR-132 in livers of HFD-feeding (**a**), HCD-feeding (**b**) and *ob/ob* mice (**c**) as well as control mice, respectively. All data are shown as mean ± s.e.m.. * *p*<0.05, ** *p*<0.01 by unpaired two-tailed student’s *t*-test.

**
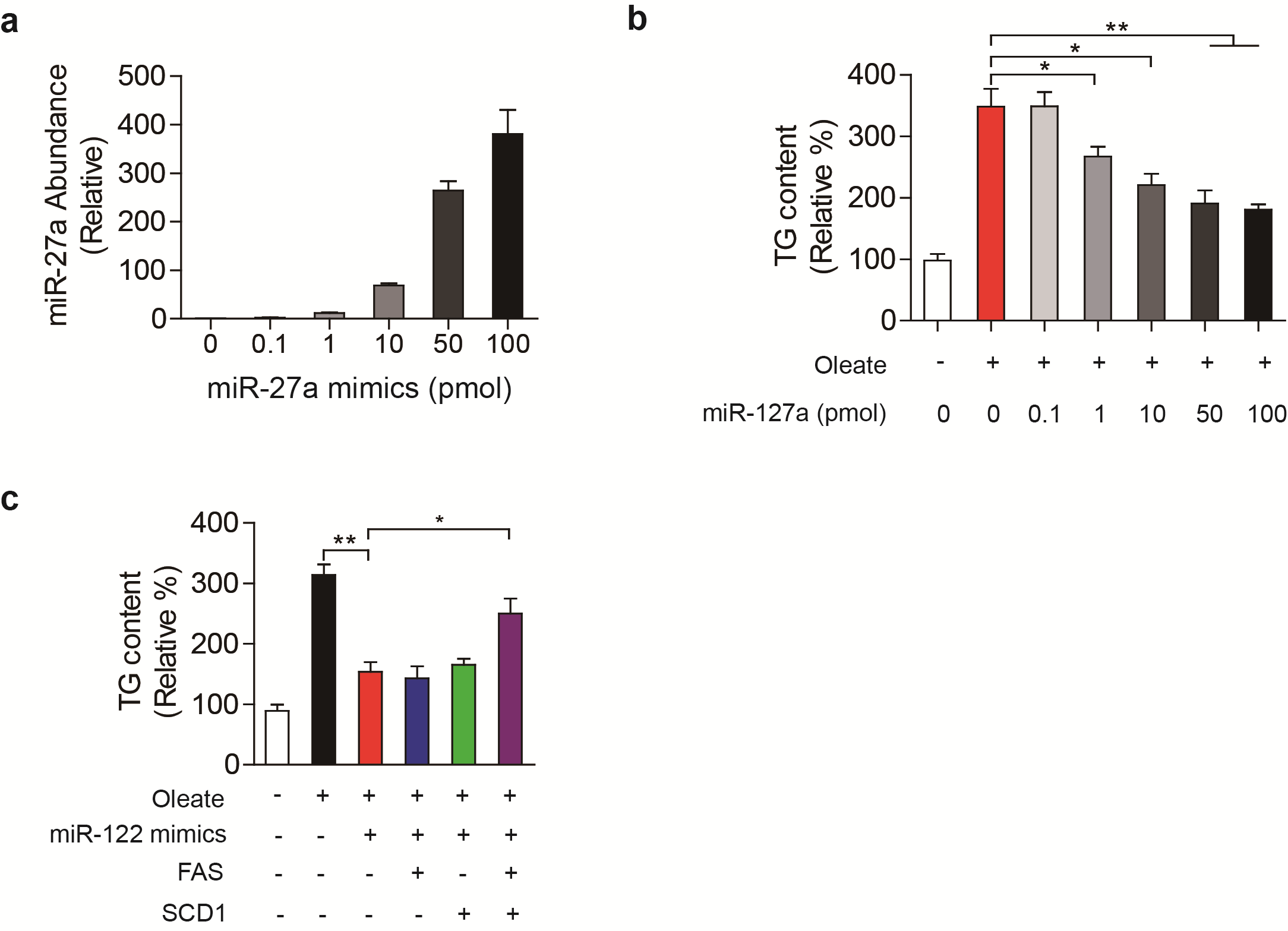
**

**Supplementary Figure 2. Related to Figure 2. a**, MiR-27a levels in mouse primary hepatocytes transfected with serial volumes of miR-27a mimics. The numbers indicate the volume of mimics added into each well of cells cultured in 24-well plate. **b**, TG contents were determined in sodium oleate-treated hepatocytes transfected with various volumes of miR-27a mimics as described in **a**. **c**, TG contents of sodium oleate-treated primary hepatocytes which were firstly transfected with miR-122 mimics or combined with plasmids to overexpress Fasn and Scd1. All data are shown as mean ± s.e.m.. * *p*<0.05, ** *p*<0.01 by one-way ANOVA.

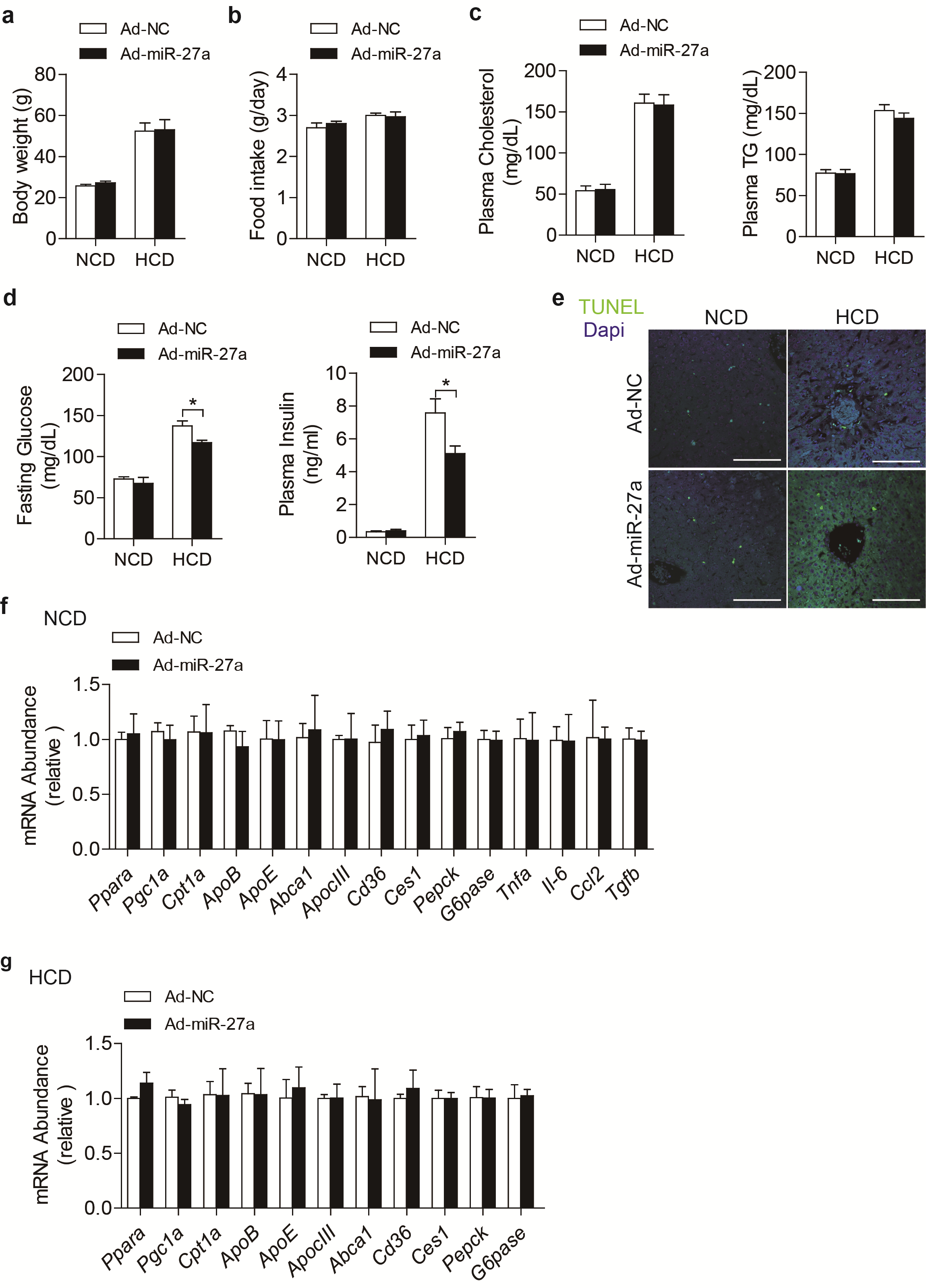


**Supplementary Figure 3. Related to Figure 3.** C57BJ/6L fed on normal chow diet (NCD, n=12 per group) or high carbohydrate diet (HCD, n=10 per group) for 8 weeks and then administrated with indicated adenovirus. **a,** Body weight. **b**, Food intake. **c,** Plasma contents of cholesterol and TG. **d,** Plasma contents of glucose and insulin. **e,** TUNEL assays of the livers. Scale bar is 100 μm. **f-g,** Real-time PCR analysis of genes involved in β-oxidation, vLDL secretion and lipid uptake, gluconeogenesis and proinflammatory and fibrigenic cytokines of livers of NCD-fed (**f**) and HCD-fed (**g**) mice. All data are shown as mean ± s.e.m.. * *p*<0.05 by unpaired two-tailed student’s *t*-test or two-way ANOVA.


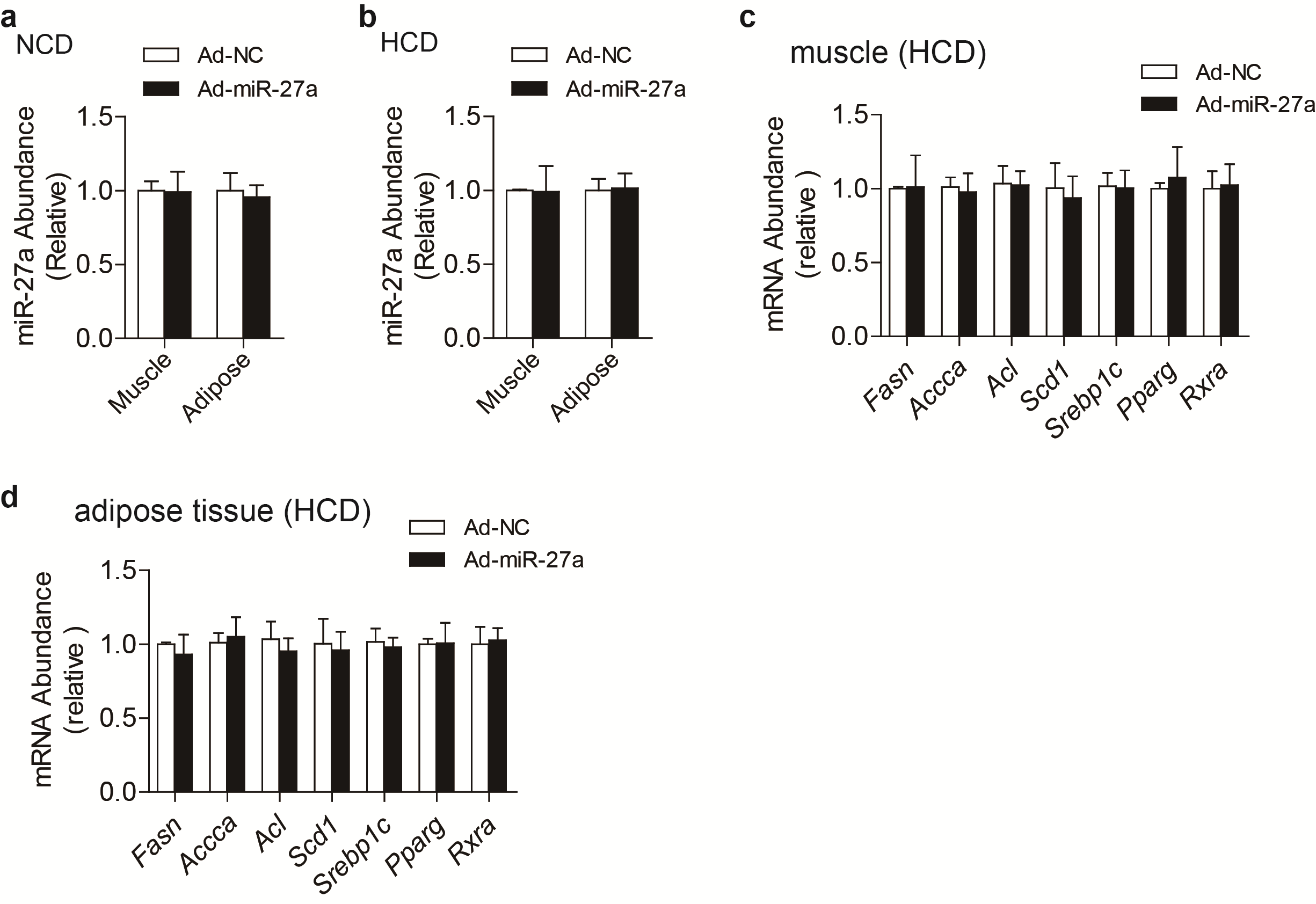


**Supplementary Figure 4. Adenovirus administration displays no effects on expression of miR-27a and lipogenesis-associated genes in skeletal muscle and adipose tissue of HCD-fed mice.** C57 BL/6 mice were fed on HCD for 8 weeks and then administered with indicated adenovirus (n=10 per group). **a-b,** MiR-27a levels of skeletal muscle and epididymal white adipose tissue in NCD-feeding mice (**a**) and HCD-feeding mice (**b**). **c-d,** Real-time PCR analysis of genes involved in lipogenesis in skeletal muscle (**c**) and epididymal white adipose tissue (**d**). All data are shown as mean ± s.e.m..


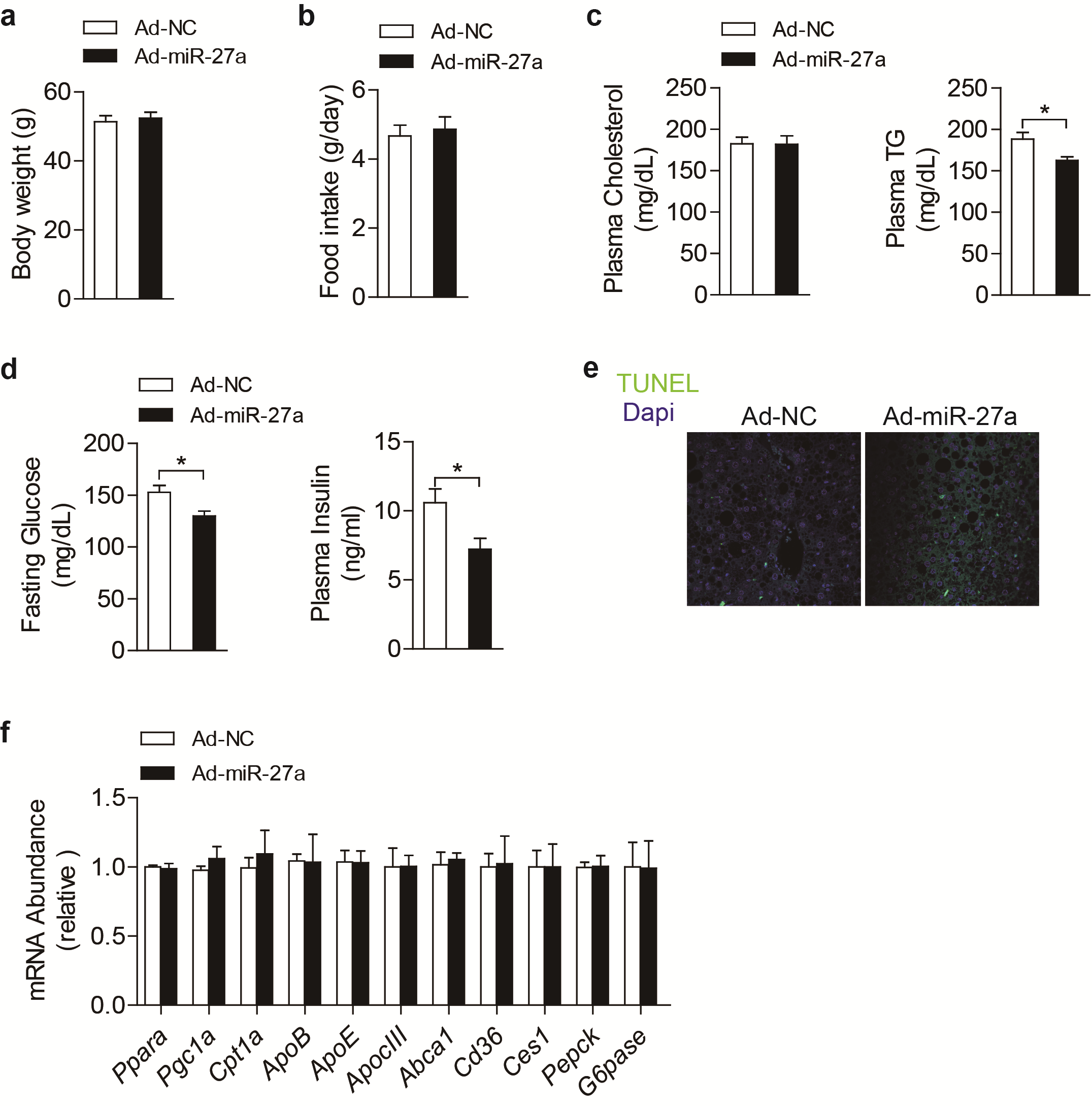


**Supplementary Figure 5. Related to Figure 4.** 16-week-old *ob/ob* mice were administered with indicated adenovirus (n=8 per group). **a,** Body weight. **b**, Food intake. **c,** Plasma contents of cholesterol and TG. **d,** Plasma contents of glucose and insulin. **e,** TUNEL assays of the livers. Scale bar is 100 μm. **f,** Real-time PCR analysis of genes involved in β-oxidation, vLDL secretion, lipid uptake and gluconeogenesis in livers. All data are shown as mean ± s.e.m.. * *p*<0.05 by unpaired two-tailed student’s *t*-test.


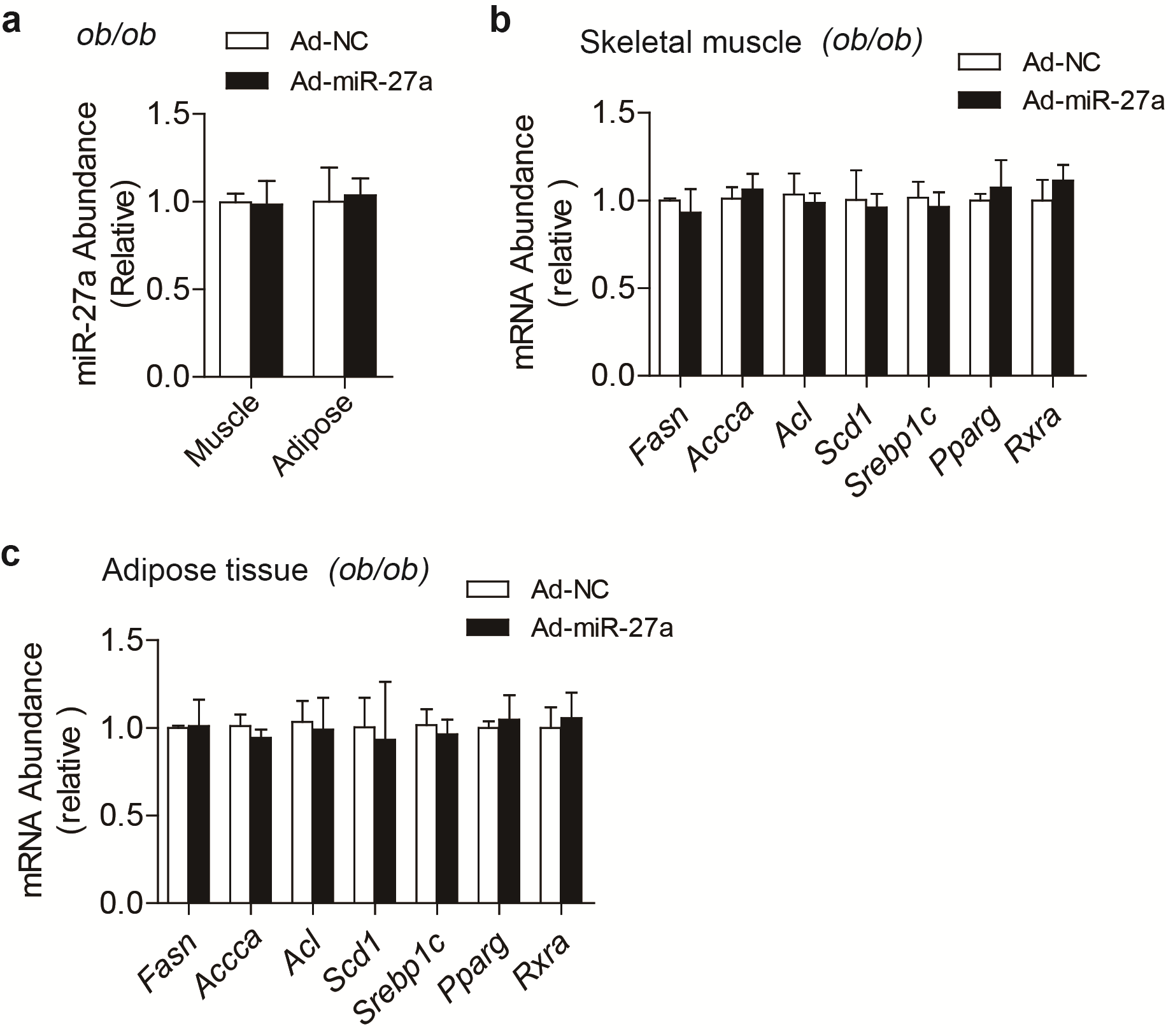


**Supplementary Figure 6. Adenovirus administration displays no effects on expression of miR-27a and lipogenesis-associated genes in skeletal muscle and adipose tissue of *ob/ob* mice.** 16-week-old *ob/ob* mice were administrated with indicated adenovirus (n=8 per group). **a,** MiR-27a levels of skeletal muscle and epididymal white adipose tissue. **b-c,** Real-time PCR analysis of genes involved in lipogenesis of skeletal muscle (**b**) and epididymal white adipose tissue (**c**). All data are shown as mean ± s.e.m..


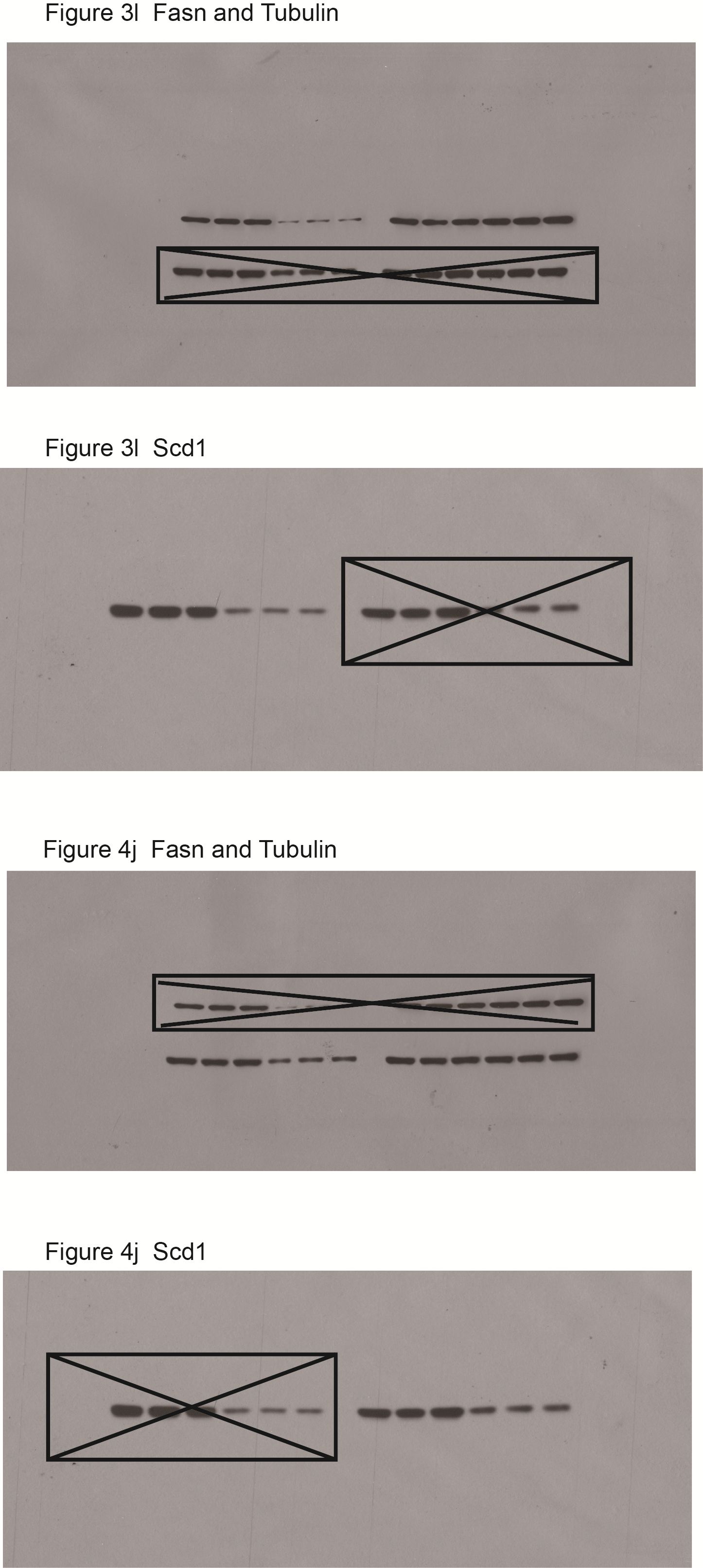


**Supplementary Figure 7. Full-length blots for Figure 3l and Figure 4j.**
